# Longitudinal “Real-World” Outcomes of Pirfenidone in Idiopathic Pulmonary Fibrosis in Greece

**DOI:** 10.3389/fmed.2017.00213

**Published:** 2017-11-29

**Authors:** Argyrios Tzouvelekis, Theodoros Karampitsakos, Paschalis Ntolios, Vasilios Tzilas, Evangelos Bouros, Evangelos Markozannes, Ioanna Malliou, Aris Anagnostopoulos, Andreas Granitsas, Paschalis Steiropoulos, Katerina Dimakou, Serafeim Chrysikos, Nikolaos Koulouris, Demosthenes Bouros

**Affiliations:** ^1^First Academic Department of Pneumonology, Hospital for Diseases of the Chest “Sotiria”, Medical School, National and Kapodistrian University of Athens, Athens, Greece; ^2^Division of Immunology, Biomedical Sciences Research Center “Alexander Fleming”, Athens, Greece; ^3^5th Respiratory Department, Hospital for Diseases of the Chest “Sotiria”, Athens, Greece; ^4^Department of Pneumonology, University Hospital of Alexandroupolis, Democritus University of Thrace, Komotini, Greece

**Keywords:** pirfenidone, safety, efficacy, idiopathic pulmonary fibrosis, treatment

## Abstract

**Background:**

Pirfenidone is an antifibrotic compound able to slow down disease progression in patients with idiopathic pulmonary fibrosis (IPF).

**Objective:**

To investigate the safety and efficacy of pirfenidone in patients with IPF in a real-life setting.

**Methods:**

This was a multicenter, retrospective, real-life, observational study for patients with IPF receiving pirfenidone.

**Results:**

We identified 92 patients with IPF receiving pirfenidone. Eighty patients (70 males and 10 females, mean age ± SD: 68.1 + 7.5, mean %FVC ± SD = 74.9 ± 17.2, mean %DL_CO_ ± SD = 48.1 ± 16.9) were included in the analysis. Skin-related (25%) and gastrointestinal (17.5%) adverse events were the most common and led to drug discontinuation in 22.5% of cases. The majority (87%) of patients experienced side effects during the first 6 months of treatment. At 36 months, changes in %FVC and %DL_CO_ were −9.25 ± 16.34 and −9.26 ± 15.26, respectively. At 6, 12, and 24 months after treatment initiation (*n* = 80, 60, and 26), 18, 15, and 5 patients (22.5, 25, and 19.2%) experienced significant (>10%) and 11, 3, and 3 patients (13.8, 5, and 11.5%) experienced marginal (5–10%) %FVC improvement; and 13, 6, and 1 patient (16.2, 10, and 3.9%) experienced marginal (−5 to −10%) and 20, 21, and 8 patients (25, 35, and 30.8%) experienced significant decline (<−10%) in %FVCpred. Median survival was 851 days, and 41 patients died during the study period.

**Conclusion:**

Pirfenidone demonstrated an acceptable safety and therapeutic profile in patients with IPF on a longitudinal basis. Prospective observational registries are urgently needed to provide a real-world view of outcomes of pirfenidone in clinical practice.

## Introduction

Idiopathic pulmonary fibrosis (IPF) represents a chronic, debilitating lung disease of unknown origin, characterized by irreversible loss of lung function due to lung scarring (1). The clinical course is largely unpredictable and periods of transient clinical stability might be observed; yet, continued progression of the disease is inevitable. The median survival without lung transplantation is close to 3 years, rendering this disease the non-lung cancer disease with the gravest prognosis (2). Due to its appreciable heterogeneity and elusive pathogenesis, IPF treatment still represents an unmet need and a major challenge for both clinicians and researchers (3–9).

Until recently, lung transplantation was the only therapeutic approach with significant impact on survival (10, 11). Four years ago FDA approved two novel anti-fibrotic compounds, pirfenidone and nintedanib, which are able to reduce disease progression in large multicenter clinical trials with IPF patients (9). Pirfenidone was the first drug to be approved for the treatment of IPF in the European Union in 2011 (12). Pirfenidone represents an oral pyridine with anti-fibrotic, anti-inflammatory, and anti-oxidant properties in experimental models of lung fibrosis (13–15); yet, its exact mechanism of action is currently unknown (16, 17). Importantly, pirfenidone has been clinically evaluated and has shown beneficial effects in patients with IPF in five randomized controlled trials comprising 1,710 patients (18–20). In particular, it has been demonstrated to slow down functional deterioration and reduces the risk of death by 48% at 1 year in a prespecified pooled analysis including data from three independent cohorts of patients with IPF (18, 19). In addition, a recent study has shown that pirfenidone significantly reduced respiratory-related hospitalizations indicating a beneficial impact on acute exacerbations of the disease (21). Major treatment-related adverse events included nausea, respiratory tract infections, photosensitivity, and diarrhea (22).

Although phase 3 clinical trials are fundamental for drug approval and commercial availability, they are conducted outside the naturalistic clinical setting and quite often leave a significant proportion of patients who are seen in real-life clinical practice and may benefit from drug administration. Toward this direction and considering that pirfenidone trials were characterized by high screening failure rates (64%), several real-world observational studies in small cohorts of patients with IPF reported encouraging safety and efficacy data (23–30); yet, most of the studies were underpowered and limited by short-term follow-up periods. Recently, an open-label extension study (RECAP) reported longitudinal outcomes of pirfenidone in a large cohort of patients with IPF, previously enrolled in phase 3 trials, further reinforcing the beneficial profile of pirfenidone (31); yet, the study was hampered by inherent weaknesses of an extension study including selection bias (32).

To this end, we aimed to report for the first time the longitudinal safety and efficacy outcomes of pirfenidone in patients with IPF derived from multiple clinical centers in Greece.

## Materials and Methods

Between September 2011 and September 2016, patients with IPF who completed at least 6 months treatment with pirfenidone (2,403 mg/day) were included in this analysis. Diagnosis of IPF was based on ATS/ERS guidelines (1). Retrospective data analysis was approved by the institutional review board of Hospital for Diseases of the Chest “Sotiria”, Medical School, and University of Athens, Greece (3876/21-2-2017). Patients were informed for known adverse events of pirfenidone and were instructed to avoid exposure to sunlight and alcohol consumption. Laboratory tests including complete blood count (CBC), renal and liver panels were performed before administration of the compound and also at monthly intervals for the first three months after the initiation of treatment and once every 3 months afterward. Patients underwent pulmonary function tests (PFTs), including body plethysmography and single breath test for determination of lung volumes and diffusing capacity of the lung for carbon monoxide (DL_CO_), during the period of diagnosis, as well as 6, 12, 24, and 36 months post treatment initiation. Combined pulmonary fibrosis and emphysema (CPFE) was defined as the presence of emphysematous lesions in >10% of the affected lungs. Pulmonary hypertension was defined as right ventricle systolic pressure (RVSP) > 35 + central venous pressure (CVP) measured by echocardiography. Continuous data were recorded as medians with ranges or mean ± SD. Independent samples *t*-tests were used to assess statistical significance between changes in %FVC (%ΔFVC) and %DL_CO_ (%ΔDL_CO_). Furthermore, patients were divided based on changes in %FVC in the following groups: significant improvement (≥10%), marginal improvement (5–10%), stability (−5 to 5%), marginal decline (−5 to −10%), and significant decline (≤−10%). Finally, a subgroup analysis of patients receiving pirfenidone for 5 years was performed.

## Results

### Baseline Characteristics

The baseline characteristics of patients involved in this study are summarized in Table 1. Between September 2011 and September 2016, we identified 92 patients with IPF. Twelve were excluded from the analysis due to either less than 6 months treatment (*n* = 5) or inconclusive diagnosis (possible UIP pattern in HRCT, *n* = 7). We included 80 patients (70 males and 10 females) of mean age ± SD: 68.1 + 7.5 years and mild-to-moderate disease severity (mean %FVC ± SD = 74.9 + 17.2 and mean %DL_CO_ ± SD = 48.1 + 16.9). Most patients (74%) were ex-smokers (*n* = 59), 17 patients were current smokers, while four patients had never smoked. Eleven patients (13.8%) underwent video-assisted thoracoscopic surgery (VATS) for lung biopsy which was consistent with UIP pattern in all cases. CPFE was present in 24 cases (30%). PH was identified in 19 patients (23.75%). Gastro-esophageal reflux (GER) and arterial hypertension were present in 39 patients (48.75%). Fourteen patients (17.5%) had hypercholesterolemia. Eight of the patients (10%) reported symptoms suggestive of an underlying connective tissue disorder, including Raynaud (*n* = 6), mild arthralgia (*n* = 3), and/or myalgia (*n* = 2), and 10 patients (13%) had positive antinuclear antibodies (ANA > 1/160); yet, the rest specific circulating auto-antibodies (extractable nuclear antigen-ENA panel, rheumatoid factor, cyclic citrullinated peptides-CCPs) were negative. Eight patients (10%) experienced occupational exposures to metal, wood, dust, or solvents. All patients were naïve of treatment prior pirfenidone treatment and none of the patients were under concomitant anti-inflammatory, immunomodulatory, or anti-fibrotic agents together with pirfenidone use. Median latency period between time of diagnosis and treatment initiation was 155 days (95% CI: 120–213).

**Table 1:** Baseline characteristics of studied population.

Characteristics	Baseline data, *n* (%)
Total patients enrolled	80
Male/female	70/10
Age (mean years ± SD)	68.1 ± 7.5
Never smokers	4 (5)
Current smokers	17 (21.3)
Ex-smokers	59 (73.8)
VATS	11 (13.8)
Prior treatment	0
CPFE	24 (30)
GERD	39 (78)
AH	39 (78)
Hypercholesterolemia	14 (17.5)
PH (Echo-RVSP > 35 mmHg)	19 (23.8)
FVC%pred (mean ± SD)	74.9 ± 17.2
DL_CO_%pred (mean ± SD)	48.1 ± 16.9
GAP score (median)	3

*AH, arterial hypertension; CPFE, combined pulmonary fibrosis and emphysema; DL_CO_, diffusion capacity of lung for carbon monoxide; FVC, forced vital capacity; GAP, gender; age and physiology; GERD, gastroesophageal reflux disease; 6MWT, 6-minute walk test; PH, pulmonary hypertension; RVSP, right ventricle systolic pressure; TLC, total lung capacity; VATS, video-assisted thoracic surgery*.

### Efficacy

**Figure 1:**
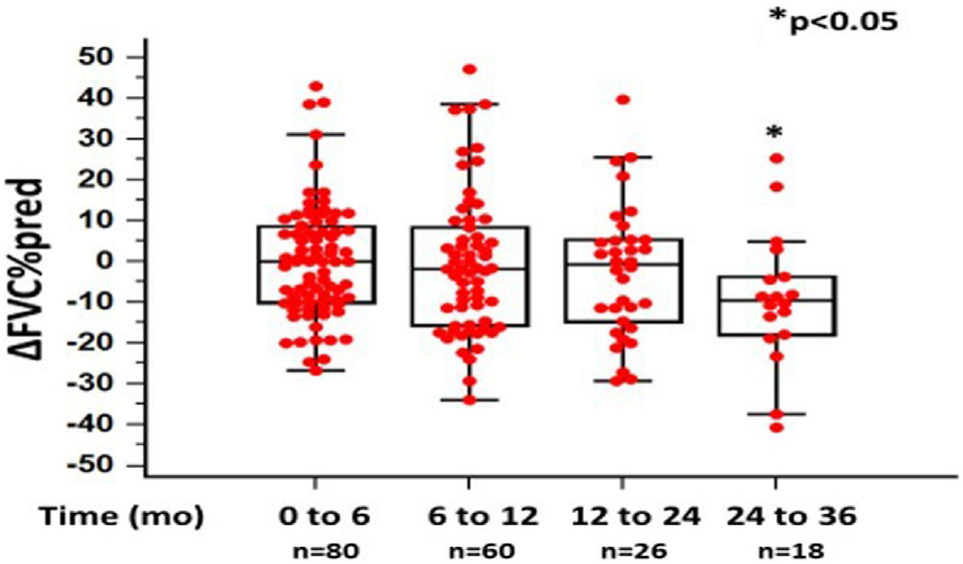
Changes in %forced vital capacity (ΔFVC) as %predicted ± SD, at different time points following pirfenidone treatment. Time 0 denotes the onset of treatment. Deaths were treated as censored. One-way ANOVA, *p* < 0.05.

**Figure 2:**
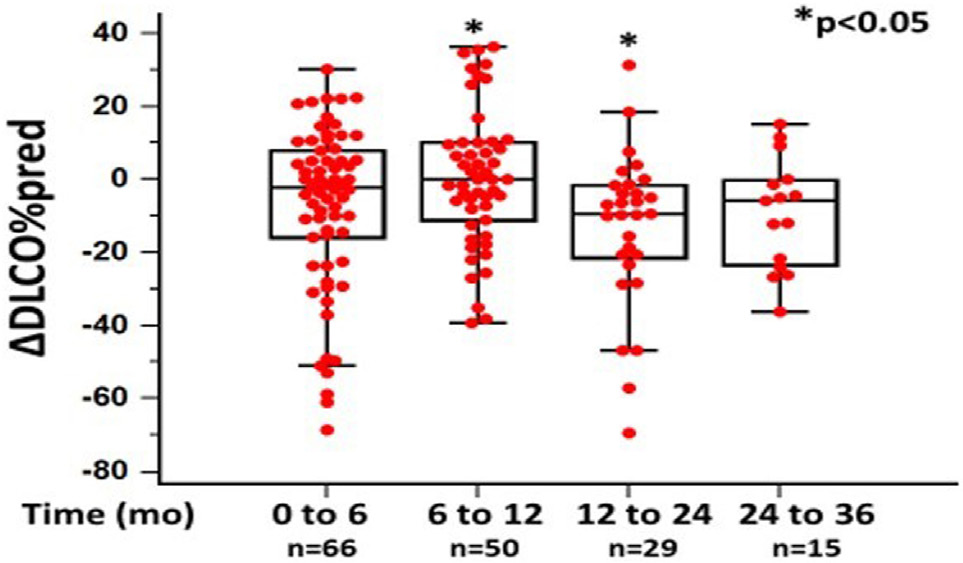
Changes in %diffusion capacity of lung for carbon monoxide (ΔDL_CO_) as %predicted ± SD, at different time points following pirfenidone treatment. Time 0 denotes the onset of treatment. Deaths were treated as censored. One-way ANOVA, *p* < 0.05.

In the 6-month follow-up, changes in %FVC and %DL_CO_ were 0.14 ± 14.04 and −6.75 ± 23.53, respectively (*n* = 80). Between 6 and 12 months, mean changes in %FVC and %DL_CO_ were −0.61 ± 17.48 (*p* = 0.068) and 3.5 ± 26.09 (*p* < 0.05), respectively (*n* = 60). Between 12 and 24 months, mean change in %FVC was −2.40 ± 16.13 (*p* = 0.624), while mean change in DL_CO_ was −13.3 ± 21.45 (*p* < 0.05) (*n* = 26). Finally, between 24 and 36 months, mean changes in %FVC and %DL_CO_ were −9.2575 ± 16.34 (*p* < 0.05) and −9.2% ± 15.26 (*p* = 0.520) (*n* = 18), respectively (Figures 1 and 2; Table 2).

**Table 2:** Changes in %ΔFVC and %ΔDL_CO_ as %predicted ± SD, at different time points (6, 12, 24, and 36 months) following pirfenidone treatment, one-way ANOVA and independent samples *t*-test, *p* < 0.05.

	0–6 months	6–12 months	12–24 months	24–36 months	*p*_6–12_	*p*_12–24_	*p*_24–36_
%ΔFVC	0.14 ± 14.04	−0.61 ± 17.48	−2.40 ± 16.13	−9.25 ± 16.34	0.068	0.624	**<0.05**
%ΔDL_CO_	−7.46 ± 22.4	0.32 ± 18.7	−13.30 ± 21.45	−9.26 ± 15.26	**<0.05**	**<0.05**	0.520

*DL_CO_, diffusion capacity of lung for carbon monoxide; FVC, forced vital capacity; GAP, gender; age and physiology*.

**Figure 3:**
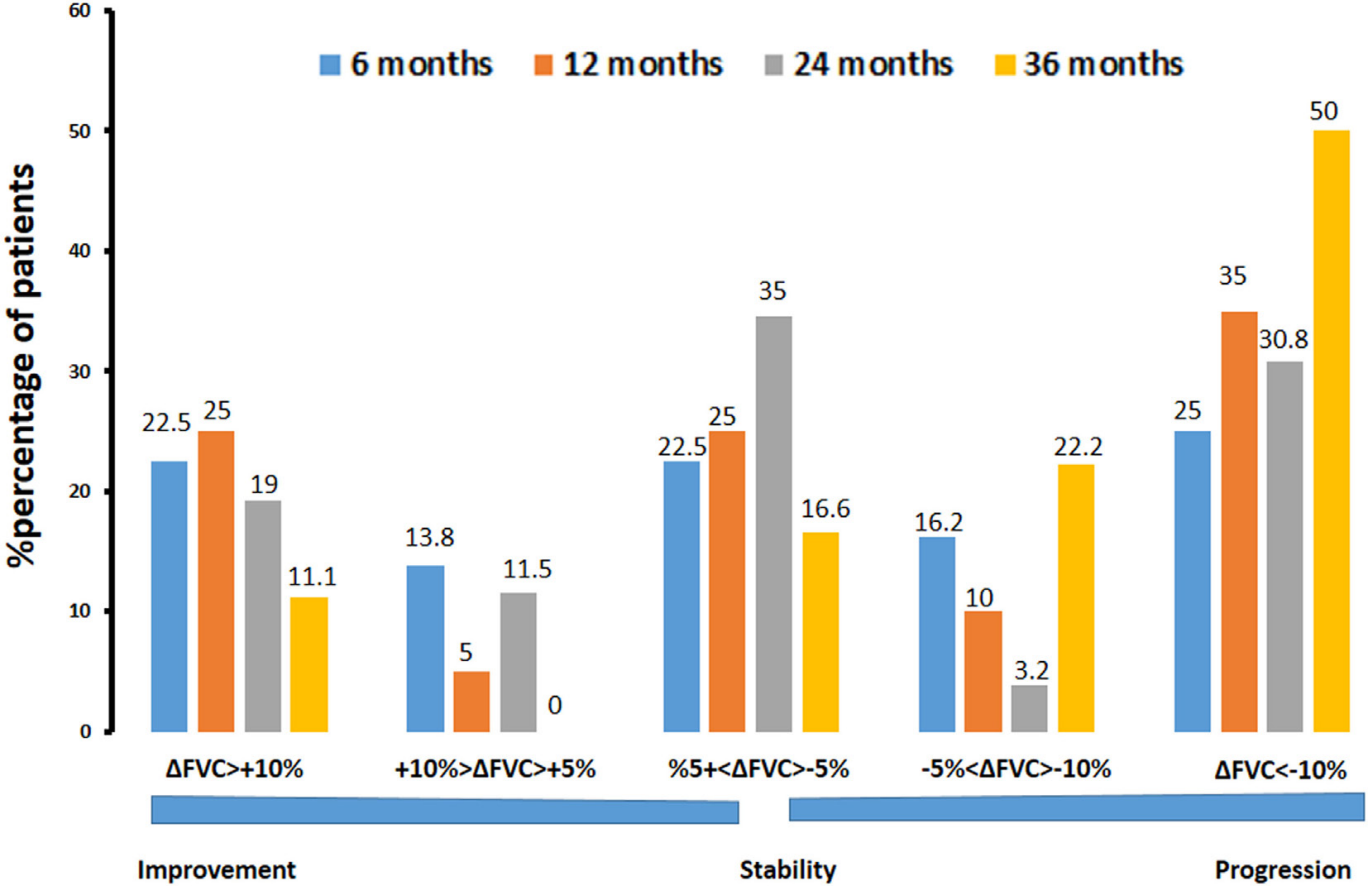
Percentage of patients with IPF experiencing significant (>10%) or marginal (5–10%) improvement, stability (−5 to 5%), and marginal (−5 to −10%) or significant (<−10%) decline in %forced vital capacity (%ΔFVC) at different time points (6, 12, 24, and 36 months) following pirfenidone treatment. Note that 65% of patients experienced disease stability after 24 months of treatment.

Further subgroup analysis revealed that 6 months after commencing pirfenidone (*n* = 80), 18 patients (22.5%) experienced significant (>10%) and 11 patients (13.8%) experienced marginal (5–10%) %FVC improvement. In addition, 18 patients (22.5%) experienced stability (−5 to 5%), 13 patients (16.2%) showed marginal (−5 to −10%), and 20 patients (25%) showed significant decline (<−10%) in %FVC predicted. At 12 months (*n* = 60), 15 patients (25%) presented with significant (>10%) and three patients (5%) experienced marginal (5–10%) %FVC improvement. Furthermore, 15 patients (25%) experienced stability (−5 to 5%), 6 patients (10%) showed marginal (−5 to −10%), and 21 patients (35%) showed significant decline (<−10%) in %FVC predicted. At 24 months (*n* = 26), five patients (19.2%) experienced significant (>10%) and three patients (11.5%) experienced marginal (5–10%) %FVC improvement. Moreover, nine patients (34.6%) presented with stability (−5 to 5%), 1 patient (3.9%) experienced marginal (−5 to −10%), and 8 patients (30.8%) showed significant (<−10%) decline in % FVC predicted (Figure 3).

There was no correlation between latency period and changes in %FVC and %DL_CO_ at 6, 12, and 24 months following pirfenidone treatment. There were also no significant differences in changes in %FVC and % DL_CO_, at 6, 12, and 24 months following pirfenidone treatment in patients with latency period below compared to those above the median cutoff threshold (155 days) (data not shown).

With regard to mortality, 41 patients (51.25%) died during the study period (1,825 days). Median survival was 851 days. Relative 1- and 3-year mortality rates were 1.25 and 32.5%, respectively (Table 3).

**Table 3:** Main adverse events during the treatment period.

Adverse event	*N* (%) (total = 80)
Photosensitivity/rash	20 (25)
Gastrointestinal	15 (18.8)
Liver toxicity	6 (7.5)
Nausea	6 (7.5)
Other	2 (2.5)
Discontinuation	18 (22.5)
Photosensitivity/rash	9 (11.2)
Gastrointestinal	6 (7.5)
Liver toxicity	4 (5)
Other	2 (2.5)

### Safety

Pirfenidone exhibited an acceptable safety profile similar to that reported in the open-label extension study (RECAP) as well as the three parent phase 3 clinical trials (CAPACITY 1 and 2, ASCEND) (Table 4). Skin-related adverse events (25%) and gastrointestinal disorders (18.8%) were the most commonly reported adverse events. A total of 18 patients (22.5%) had to permanently discontinue pirfenidone due to severe adverse events including skin-related adverse events (*n* = 9), gastrointestinal disorders (*n* = 6), liver toxicity (*n* = 4), tibial edema (*n* = 1), and anal fistula (*n* = 1). Among these patients, three presented with both skin-related and gastrointestinal adverse events. Collectively, 89% (*n* = 71) of patients reported at least one side effect and 22.5% (*n* = 18) had to permanently discontinue pirfenidone. The majority of patients experienced side effects of mild severity (*n* = 62/71, 87%) or had to permanently discontinue pirfenidone due to severe side effects (*n* = 13/18, 72%) during the first 6 months of treatment. Drug retention rates (including patients who discontinued drug due to death) were 83, 76, 51, and 45% at 6, 12, 24, and 36 months, respectively. Median drug exposure time was 20 months.

**Table 4:** Mortality data of studied population.

Parameter	Number (absolute or relative)
Number of patients	80
Study period (days)	1,825
Median survival (days)	851
Deaths during study period	41 (51.25%)
1-year absolute mortality	1
1-year relative mortality	1.25%
3-year absolute mortality	26
3-year relative mortality	32.5%

## Discussion

This is the first study in Greece that provides longitudinal outcomes on safety and effectiveness of pirfenidone in a moderately sized cohort of patients with IPF over a study period of 5 years. Our safety and efficacy data are in line with those reported in the large open-label extension RECAP study as well as in small observational real-world retrospective studies. In addition, we demonstrate that a large percentage of patients with IPF receiving pirfenidone experience functional improvement while functional stabilization is sustained even after 3 years of treatment.

Pirfenidone was the first approved as anti-fibrotic compound for the treatment of IPF (28). Current recommendations suggest the use of pirfenidone for patients with IPF and relatively preserved lung volumes (33). Therefore, we present our real-life clinical experience on the safety and efficacy profile of this compound in a cohort of 80 patients with IPF and mild-to-moderate functional impairment. Our study exhibits several attributes considering that it adds significant knowledge on long-term pirfenidone safety and effectiveness profile within the naturalistic clinical setting. In particular, our study overcomes inherent weaknesses of phase 3 clinical trials including inability to map drug risks within the real-world clinical practice and generalizability of presented results in all subgroups of patients with IPF irrespective of disease severity.

The adverse events recorded in our study were consistent with the already known safety profile of pirfenidone, including skin-related disorders, gastrointestinal disorders, and liver toxicity (34, 35). Interestingly, the percentage of skin-related adverse events (25%) was higher in Greece than those reported in other real-life studies of northern countries with different climate, including United Kingdom and Belgium–Netherlands (10.3 and 15.8%, respectively) (24, 29). The percentage of gastrointestinal disorders (18.8%) was comparable to that reported in RECAP (22.9%). Furthermore, in our study, 89% of patients reported at least one side effect, which is comparable with the 98% reported in RECAP (31, 36,–37). Adverse events led to discontinuation in 22.5% of our patients, which is a slightly higher percentage than those reported in ASCEND (14.4%) (18), CAPACITY (15%) (19), and RECAP (11.3%) studies (31, 36, 37). In our center, skin-related and gastrointestinal disorders were also common causes leading to drug discontinuation, while there were also four cases of drug discontinuation due to liver toxicity. Interestingly, two patients discontinued pirfenidone due to tibial edema and anal fistula, respectively.

With regard to functional indices, our baseline values (mean%FVC = 74.9 and mean%DL_CO_ = 48.1) are comparable to those reported in RECAP study (mean%FVC = 70.9 and mean%DL_CO_ = 41.2). In the 6-month follow-up, changes in %FVC and % DL_CO_ were 0.14 ± 14.04 and −6.75 ± 23.53, respectively. Although there was no pretreatment period to compare, these results reinforce pirfenidone therapeutic profile in the naturalistic clinical setting (18, 24–27, 29).

Considering disease heterogeneity and the close association between FVC and mortality (38, 39), we performed a subgroup analysis of the study population based on %FVC alterations over the study period. We showed that a substantial proportion of patients with IPF (20%) receiving pirfenidone experienced significant improvement in %FVC, which sustained even after two years of treatment. This observation is in line with other observational real-world studies (23, 25, 40). Moreover, in our study, pirfenidone administration led to disease stability in 65% of patients after 24 months of treatment. These findings clearly demonstrate a beneficial safety and efficacy profile for pirfenidone even after prolonged exposure. In line with this notion, in a real-world observational study by Bando et al., pirfenidone prevented FVC decline in over half of the cases after 24 months of treatment (23). Importantly, in our study, therapeutic effects were also sustained after 3 years of treatment, as indicated by functional stabilization in almost half of the patients. Finally, we have demonstrated similar mortality rates to those reported in phase 3 clinical trials (1-year mortality 1.25 versus 3.5%, *p* = 0.52) (22) as well as in the RECAP study (32.5% 3-year mortality versus 21.8% 5-year mortality, *p* = 0.11). This observation adds a significant strength to pirfenidone efficacy profile on a longitudinal basis and validates pivotal evidence in the everyday clinical setting.

Our study exhibited several limitations that should be treated cautiously. First, this was a retrospective, observational study and thus it presents with inherent weaknesses of this type of analysis. Second, there was no pretreatment period to compare rates of functional decline following pirfenidone administration. This can be explained by limitations of the pragmatic clinical environment considering that the majority of patients were newly diagnosed or did not have available functional data. Third, this was an underpowered study compared to large randomized controlled trials; yet, its size is acceptable for an observational real-world study.

In conclusion, this is the first real-world observational study reporting longitudinal outcomes of pirfenidone use in patients with IPF of mild-to-moderate disease severity in Greece. Our study clearly demonstrates an acceptable long-term safety and efficacy profile, which is sustained even after 3 years of treatment. Our observations reinforce earlier findings from small observational real-world studies as well as data derived from the large open-label extension RECAP study. The identification of a substantial minority of patients experiencing functional improvement following pirfenidone administration is of cardinal importance. Pharmacogenomic analyses and prospective real-world registries are urgently needed to address residual questions of safety and efficacy of pirfenidone in different endotypes of patients with IPF and identify those that will benefit the most.

## Ethics Statement

Retrospective data analysis was approved by the institutional review board of Hospital for Diseases of the Chest “Sotiria”, Medical School, and University of Athens, Greece (3876/21-2-2017).

## Author Contributions

All the authors contributed to data interpretation and acquisition, article draft, revision, and final approval.

## Conflict of Interest Statement

The authors declare that the research was conducted in the absence of any commercial or financial relationships that could be construed as a potential conflict of interest.
