# Corrigendum: Longitudinal “Real-World” Outcomes of Pirfenidone in Idiopathic Pulmonary Fibrosis in Greece

**DOI:** 10.3389/fmed.2017.00257

**Published:** 2018-01-24

**Authors:** Argyrios Tzouvelekis, Theodoros Karampitsakos, Paschalis Ntolios, Vasilios Tzilas, Evangelos Bouros, Evangelos Markozannes, Ioanna Malliou, Aris Anagnostopoulos, Andreas Granitsas, Paschalis Steiropoulos, Katerina Dimakou, Serafeim Chrysikos, Nikolaos Koulouris, Demosthenes Bouros

**Affiliations:** ^1^First Academic Department of Pneumonology, Hospital for Diseases of the Chest “Sotiria”, Medical School, National and Kapodistrian University of Athens, Athens, Greece; ^2^Division of Immunology, Biomedical Sciences Research Center “Alexander Fleming”, Athens, Greece; ^3^5th Respiratory Department, Hospital for Diseases of the Chest “Sotiria”, Athens, Greece; ^4^Department of Pneumonology, University Hospital of Alexandroupolis, Democritus University of Thrace, Komotini, Greece

**Keywords:** pirfenidone, safety, efficacy, idiopathic pulmonary fibrosis, treatment

In the original article, there was a mistake in Figure 1 as published [*x* and *y* axes were mislabeled and ⋆*p*-value <0.05 indicating significance was missing]. The corrected Figure 1 appears below. The authors apologize for this error and state that this does not change the scientific conclusions of the article in any way.

**Figure 1:**
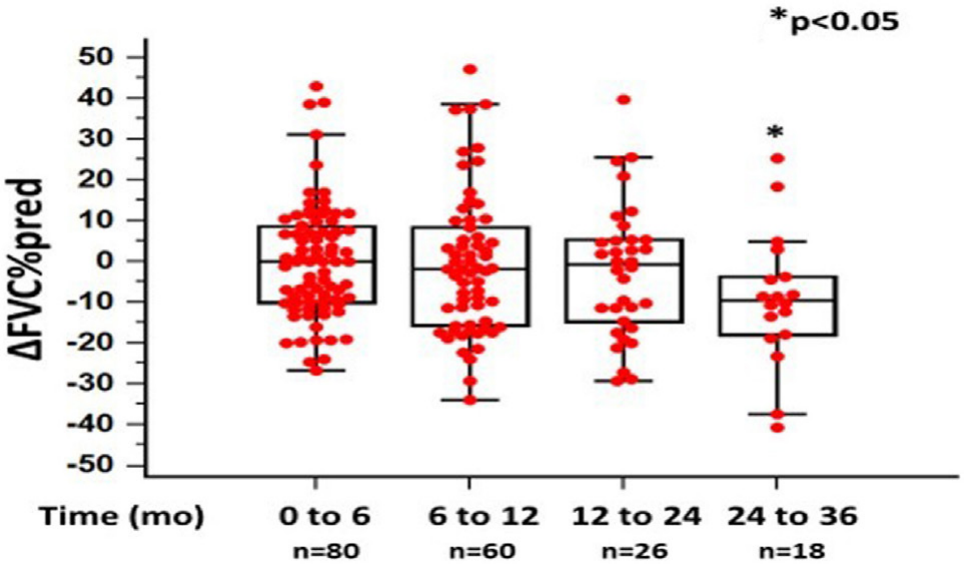
Changes in %forced vital capacity (ΔFVC) as %predicted ± SD, at different time points following pirfenidone treatment. Time 0 denotes the onset of treatment. Deaths were treated as censored. One-way ANOVA, *p* < 0.05.

In the original article, there was a mistake in Figure 2 as published [*x* and *y* axes were mislabeled and ⋆*p*-value <0.05 indicating significance was missing]. The corrected Figure 2 appears below. The authors apologize for this error and state that this does not change the scientific conclusions of the article in any way.

**Figure 2:**
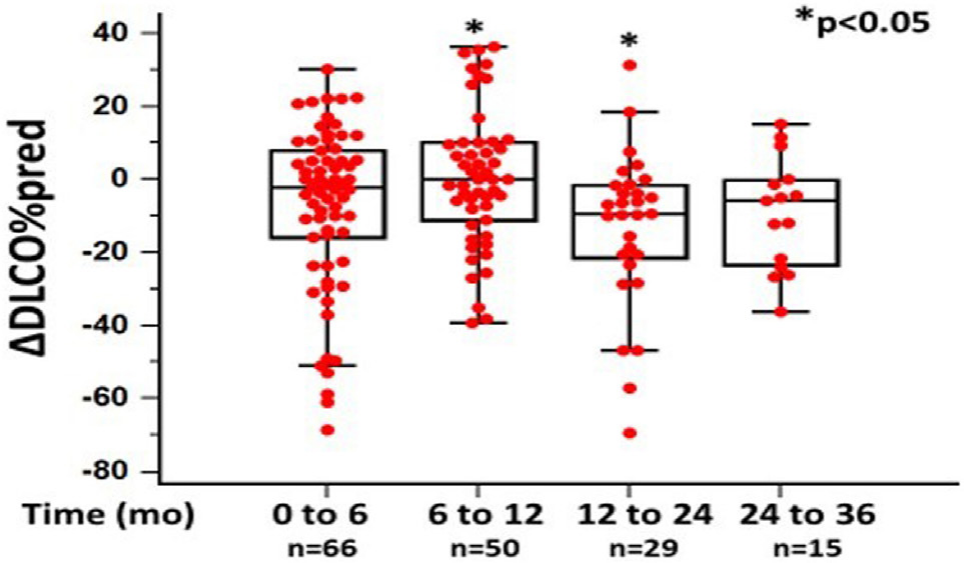
Changes in %diffusion capacity of lung for carbon monoxide (ΔDL_CO_) as %predicted ± SD, at different time points following pirfenidone treatment. Time 0 denotes the onset of treatment. Deaths were treated as censored. One-way ANOVA, *p* < 0.05.

The original article was updated.

## Conflict of Interest Statement

The authors declare that the research was conducted in the absence of any commercial or financial relationships that could be construed as a potential conflict of interest.

